# Note on the Treatment of Malignant Neoplasms by Electricity

**Published:** 1899-07

**Authors:** J. McF. Gaston

**Affiliations:** Atlanta, Ga.


### NOTE ON THE TREATMENT OF MALIGNANT NEOPLASMS BY ELECTRICITY.

BY. J. McF. GASTON, JR.,
ATLANTA, GA.

The treatment of inorganic masses by electrolysis has been more and more prominently brought to the attention of the profession of late. *The Annals of Surgery* for May has a review of *The American Year Book of Medicine and Surgery,* and the reviewer Goodrich remarks: "Of special interest to us is the department of the work devoted to surgery. . . . Interesting cases of rare forms of tumors are recorded. Some non-operative measures for treating malignant tumors are given a hearing," and one of these is by electrolysis and cataphoresis. The case reported by J. McFadden Gaston is noted, and with the renewed interest in this subject due to the experience of other observers, the method of Dr. Gaston is now prominently before the profession. The patient was a boy, aged twelve, with an abdominal tumor, which was subjected to the microscope and pronounced round-celled sarcoma. The first application of electricity was November 17, 1895, and since the disappearance of the tumor in 1897, there has been no recurrence of the growth.

Dr. R. N. Fraser, of Thamesville, Ont., saw the article of Dr. Gaston soon after it was presented to the American Surgical Association under the title of "Successful Treatment of Sarcoma with Electrolysis and Cataphoresis, Combined with the Internal use of Donovan's Solution." He undertook to carry out the same treatment, and has written his full conviction of the efficacy of the new method. He says in a letter, dated April 8, 1899 :

" I am preparing a report of my case of recurrent malignant disease which I will probably read at the approaching meeting of the Ontario Medical Association. I will give an outline of the case reported by you [Gaston], and would be glad to know the present condition of your patient; would like also to know whether other cases have been reported which would be of interest in connection with a study of this method of treatment. My patient is now in excellent health, without any appearance of disease; over fifteen months since last removal of tumor. We are still continuing treatment."

---

### INFLUENZA.

Baccelli (*Centralblatt für innere Medicin, N. Y. Med. Jour.*) recommends the following formula for use in cases that begin with severe fever and nervous symptoms:

| | | |
|---|---|---|
| ℞ | Quinine salicylate | gr. 3 |
| | Phenacetine | gr. 2¼ |
| | Camphor | gr. ⅓ |
| M. | | |

As many as six such powders may be given in twenty-four hours. When catarrhal symptoms appear, from a third of a grain to half a grain of antimony pentasulphide may be added.

